# Dysregulation of photosynthetic genes in oceanic *Prochlorococcus* populations exposed to organic pollutants

**DOI:** 10.1038/s41598-017-08425-9

**Published:** 2017-08-14

**Authors:** Maria-Carmen Fernández-Pinos, Maria Vila-Costa, Jesús M. Arrieta, Laura Morales, Belén González-Gaya, Benjamin Piña, Jordi Dachs

**Affiliations:** 1 Department of Environmental Chemistry, IDAEA-CSIC, Barcelona, Catalunya Spain; 2 Spanish Institute of Oceanography (IEO), Oceanographic Center of The Canary Islands, Santa Cruz de Tenerife, 38180 Spain; 3 Department of Global Change Research, IMEDEA-UIB-CSIC, Esporles, Mallorca Spain

## Abstract

The impact of organic pollutants on oceanic ecosystem functioning is largely unknown. *Prochlorococcus*, the most abundant known photosynthetic organism on Earth, has been suggested to be especially sensible to exposure to organic pollutants, but the sub-lethal effects of organic pollutants on its photosynthetic function at environmentally relevant concentrations and mixtures remain unexplored. Here we show the modulation of the expression of two photosynthetic genes, *rbcL* (RuBisCO large subunit) and *psbA* (PSII D1 protein), of oceanic populations of *Prochlorococcus* from the Atlantic, Indian and Pacific Oceans when exposed to mixtures of organic pollutants consisting of the non-polar fraction of a seawater extract. This mixture included most persistent organic pollutants, semivolatile aromatic-like compounds, and the unresolved complex mixture of hydrocarbons. *Prochlorococcus* populations in the controls showed the expected diel cycle variations in expression of photosynthetic genes. However, exposure to a complex mixture at concentrations only 2-fold above the environmental levels resulted in a decrease of expression of both genes, suggesting an effect on the photosynthetic function. While organic pollutant effects on marine phytoplankton have been already demonstrated at the cellular level, this is the first field study showing alterations at the molecular level of the photosynthetic function due to organic pollutants.

## Introduction

Semivolatile and hydrophobic organic pollutants (OPs) reach the global oceans by long range atmospheric transport and deposition, can affect ecosystems, and represent a vector of global change^1–5^. Once in the water column, OPs accumulate in planktonic organisms due to their hydrophobicity^4, 6–8^. Concentrations of OPs, such as polychlorinated biphenyls (PCBs) and polycyclic aromatic hydrocarbons (PAHs), are 10^4^ to 10^7^ times higher in phytoplankton than in seawater^6, 7^. It is known that phytoplankton and bacteria play key biogeochemical controls on the occurrence and fate of OPs in the marine environment^8, 9^. However, the potential effects of OPs on the major processes driving the carbon cycle are still uncharacterized. Recently, several studies showed that mixtures of organic pollutants, including those known as persistent organic pollutants (POPs), can exert a toxic effect on marine phytoplankton, reducing their abundance and viability at concentrations one to two orders of magnitude higher than those found in oceanic waters^3, 10^. Nevertheless, the influence of OPs at environmental relevant concentrations on phytoplankton ecological functions, such as photosynthesis, remains unexplored. Phytoplankton is responsible for as much as 50% of worldwide inorganic carbon fixation through photosynthesis^11, 12^, and any perturbation of this activity would have an important impact on the carbon cycle. This potential coupling of the occurrence of OPs in the oceans and oceanic photosynthesis would help elucidate the effects of chemical pollution at global scale. This constitutes an already identified, but still unquantified, vector of global change^1^.

The cyanobacterial genera *Prochlorococcus* and *Synechococcus* are responsible of 32–80% of net primary productivity (NPP) in oligotrophic oceans^13, 14^. *Prochloroccocus* dominates phytoplankton communities under highly oligotrophic conditions and stratified waters, which makes them the most abundant photosynthetic organism known. They are ubiquitous in all oceans in the latitude band from 40°S to 40°N, and in the water column from surface to 200 m depth, presenting abundances of 10^5^ cells/mL^15^. *Prochlorococcus* is classified into two genetic and physiologically different groups. The *high-light* (HL) clade comprises strains adapted to high light intensities, usually distributed in the first 100 m depth, whereas the *low-light* (LL) clade consists of strains adapted to low light intensities and usually found between 80 m and 200 m depth^15^. *Prochlorococcus* has been described as particularly sensitive to organic pollutants and to other environmental stressors, such as UV radiation or copper, at environmentally relevant levels^3, 10, 16, 17^.

The recent development of a quantitative method to assess the expression of the photosynthetic genes *rbcL* (large subunit of RuBisCO) and *psbA* (D1 protein) of *Prochlorococcus* allowed identifying specific perturbations of these genes in *Prochlorococcus* axenic cultures when exposed to sublethal levels of simple mixtures of PAHs or organochlorine pesticides (OClPs)^18^. However, PAHs and OClPs account for less than 1% of the mixture of OPs present in seawater, which includes a chromatographically unresolved complex mixture of hydrocarbons^2^, and thousands of other OPs^19^. For example, there are large diffusive inputs of semivolatile aromatic-like compounds (SALCs) to the ocean^2^ with unknown effects on the oceanic microbiome. It remains unknown whether or not complex mixtures of OPs at the ultra-trace levels found in the ocean could influence natural *Prochlorococcus* populations. In fact, not all the organic pollutants present in seawater are known, nor can they be adequately quantified^19^. Nevertheless, the influence of mixtures of OPs can be assessed by using concentrates of the non-polar fraction of the organic matter present in seawater^3^. The known compounds only explain a small fraction of the observed toxicity of fresh- and sea-waters. For example, only 1% of the observed effects produced by organic pollutants in the environment can be explained by the known and quantified compounds^20^, or less than 1% of the oxidative stress measured in continental waters has been attributed to known compounds^21^.

Our working hypothesis is that the ubiquitous complex mixture of OPs found in the ocean, with a large contribution of the unresolved complex mixture (UCM), may represent an environmental impact for oceanic organisms, such as *Prochlorococcus*, and therefore affect photosynthesis as their main ecological function.

## Results and Discusion

**Figure 1:**
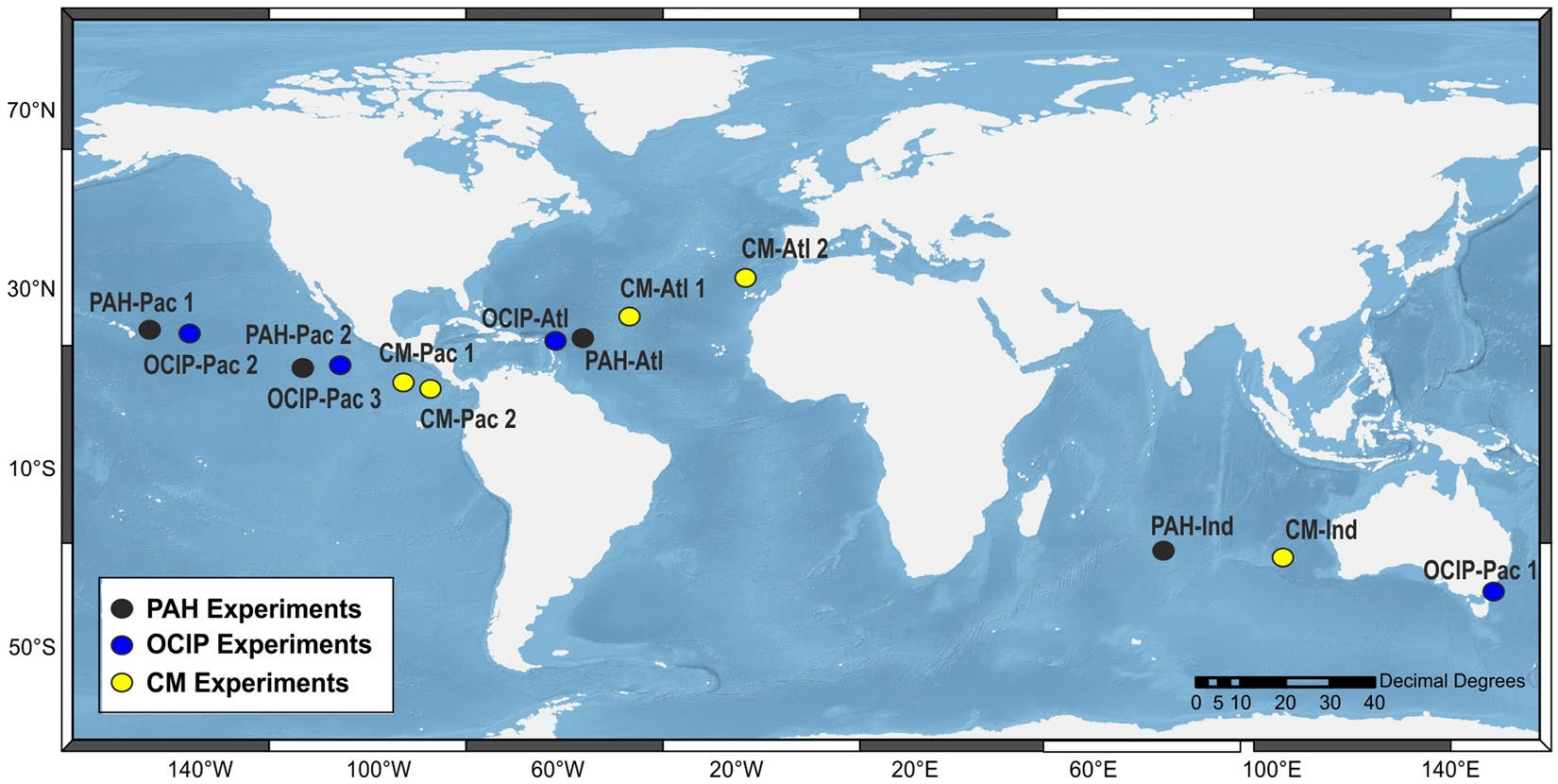
Location of the experiments. During the Malaspina circumnavigation on board of R/V Hesperides, natural communities from the deep chlorophyll maximum were challenged with the three different pollutant mixtures, polycyclic aromatic hydrocarbons (PAHs, black), organochlorine pesticides (OClP, blue), and complex chemical mixtures (CM, yellow). The map has been generated with ArcGIS, version 10.4 (www.arcgis.com).

In order to analyze the effects of both simple and complex mixtures of OPs on the photosynthetic capacity of *Prochlorococcus* at environmentally relevant levels, we quantified the expression of the two photosynthetic genes *rbcL* and *psbA* in natural communities of *Prochlorococcus* from the Atlantic, Pacific and Indian Oceans (Fig. 1) after exposure to OP mixtures. A total of 13 experiments were performed assessing the effects of a complex mixture of non-polar organic pollutants obtained from the non-polar fraction of the extracts of an oceanic seawater (see Methods), and some of the constituents of this mixture by their own, such as PAHs, and OClPs. The complex mixture contained the hydrophobic POPs present in seawater, the large SALC pool with a large contribution of the aromatic UCM^2^, and the aliphatic UCM among other OPs. As this mixture is limited to the non-polar fraction of the organic compounds, it does not contain most of the organic matter present in the dissolved phase, but only those compounds within the aliphatic and aromatic fractions (very low O/C ratio).

The measured concentrations of target OPs in the treatments were higher than in the controls for all the experiments carried out in the Atlantic, Indian and Pacific Oceans (Fig. 1, Table S1–S3). The treatments with the complex chemical mixture (CM) represented an increase of 1.3-fold in PAHs concentrations and 1.8-fold of odd-carbon number alkane concentrations (allochthonous n-alkanes)^22^. After 24 h of exposure, no significant differences were observed in the PAH concentrations (present in the CM) in comparison to initial values. Conversely, n-alkanes’ concentrations were reduced to 54% of initial concentrations (p < 0.01) (Table S3). The concentrations of OClPs and PCBs in the experiments with the CM were below the method limit of detection. The average amount of PCBs and OClPs added to 1 L of water in the treatments was of 140 pg, 8.3 pg and 3.2 pg for PCBs, HCB and HCHs, respectively. These amounts correspond to those found in 10 L of surface seawater in the NE oligotrophic Atlantic (Table S4). Therefore, the nominal increase (C/C_Control_) was 10-fold, even though the real C/C_Control_ in the incubations for other POPs, SALCs, and UCMs would have been between 1.3 and 10 times, as n-alkanes, PAHs and PCBs cover the range of properties of most POPs and hydrocarbons present in seawater^4^.

The addition of the CM to the treatments, with concentrations between 1.3 and 10 times those found in oceanic waters, did not induce a significant decrease in *Prochlorococcus*, *Synechococcus* or picoeukaryote abundances (Table S5). PAHs and OClPs are among the compounds present in the CM, and ubiquitously found in the marine environment. For the experiments where treatments were spiked with PAHs or OClPs, without the rest of the CM (Fig. 1), there was neither a significant decrease in *Prochlorococcus*, *Synechococcus* or picoeukaryote abundances (Table S5). In these experiments, PAHs and OClPs were added to treatments at concentrations significantly higher than those found in the ocean (C/C_control_ between 70 and 360 at the end of incubations for PAHs and OClPs, respectively). This lack of growth inhibition by the PAHs and the CM is consistent with the results of Echeveste and coworkers^3^, who estimated that a 10% decrease (LC10) of the *Prochlorococcus* abundance would require PAHs and CM concentrations 660 and 21 times above current oceanic levels, respectively.

**Figure 2:**
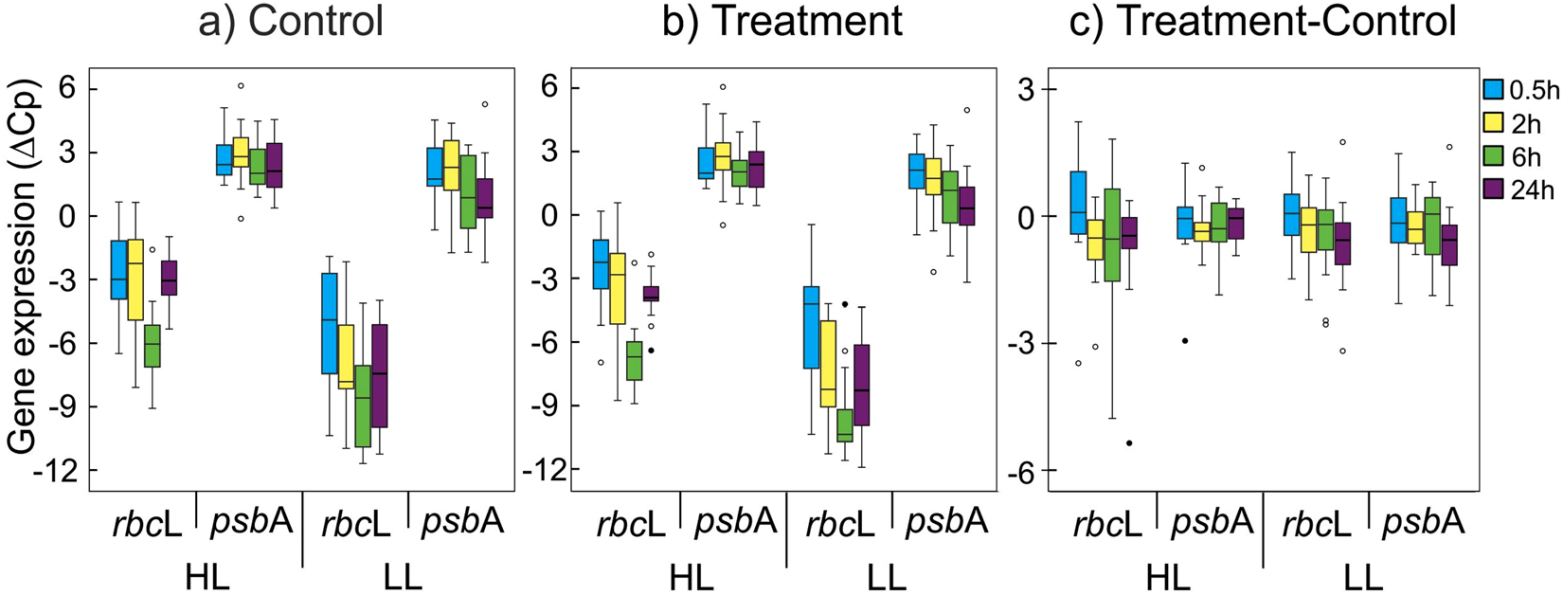
Expression time course of the photosynthetic *rbc*L and *psb*A genes. Expression of both target genes was measured for HL and LL *Prochlorococcus* separately in every sample of each experiment. This figure shows the time course of each gene in (**a**) control (no-treated) samples, (**b**) treatments (treated with any of the pollutant mixtures), and (**c**) the differential expression between treatments and controls. p values associated to the different gene expression data distributions (Kruskal-Wallis) are indicated. n/s, non-significant (p > 0.05).

Analysis of RNA abundances of the *rbcL* gene in both HL and LL clades showed the expected variations during the diel cycle^23, 24^. *rbcL* mRNA levels dropped at dusk (6 h after starting of the incubation) by almost 90% (p = 1.7*10^–3^ for Control HL- *rbcL*; p = 0.03 for Control LL- *rbcL*; p = 1.2*10^−3^ for Treatment HL- *rbcL* and p = 4.8*10^−3^ for Treatment HL- *rbcL*) compared to noon or early afternoon values (0.5, 2 and 24 h exposure times, Fig. 2a). In contrast, *psbA* mRNA values did not show significant variations (p > 0.05 for every control/treatment vs. HL/LL pair) during the same period. It has been reported that *psbA* mRNA levels show a circadian variation of about two-fold under laboratory conditions following light intensity changes^23^. However, these changes are probably too weak to be detected under our field experimental conditions, in which different strains coexist. Exposure to organic pollutants did not alter this temporal expression pattern (Fig. 2b). The diel cycle had no statistically significant effects (p > 0.05 for both HL and LL clades) on the relative expression differences of either gene between treated and non-treated (control) cultures (∆∆Cp values, Fig. 2c).

The observation of a clear diel cycle for *rbcL* expression in the field is shown here for the first time, as to the best of our knowledge, this trend has only been described before under highly controlled laboratory conditions^23, 24^. This illustrates the utility of on-board ship experiments of natural microorganism populations to allow experimental setups similar to those performed in laboratory conditions using pure cultures, but taking into account the natural genetic and community variability present in natural waters. It also confirms the quantitative nature of the methodology used to sample and analyze the expression of the photosynthetic genes.

Exposure of *Prochlorococcus* natural populations to complex mixtures of organic pollutants (CM) resulted in a decrease of *rbcL* expression for both clades (p = 0.016 for HL-*rbcL* and p = 0.025 for LL-*rbcL*, paired t-test, p < 0.05). We found temporal differences in the response of gene expression to exposure of organic pollutants. This was more evident in populations exposed to the CM, as the maximal effects occurred after 2 h of exposure (Table 1 and Figure S1). This can be described as a toxic effect, which tended to decrease as cells adapted to the presence of the mixture. In addition, the concentrations of several of the OPs present in the CM decreased during the course of the experiment, consistent with microbial degradation.

**Table 1:** Paired t-test treatment versus control for the different experiments and exposure times.

		High-light genes				Low-light genes			
		***rbc*** **L**		***psb*** **A**		***rbc*** **L**		***psb*** **A**	
		Fold Change ± sd	*p*	Fold Change ± sd	*p*	Fold Change ± sd	*p*	Fold Change ± sd	*p*
PAHs	0.5 h	1.05 ± 1.27	0.555	0.84 ± 0.77	0.145	0.94 ± 1.01	0.398	0.74 ± 0.62	0.025*
	2 h	0.78 ± 0.65	0.034*	0.91 ± 0.86	0.288	1.16 ± 1.08	0.812	1.15 ± 0.91	0.935
	6 h	1.31 ± 2.35	0.696	1.02 ± 1.05	0.546	0.70 ± 1.18	0.240	0.61 ± 0.61	0.039*
	24 h	0.78 ± 0.75	0.110	0.86 ± 0.74	0.126	0.91 ± 0.84	0.288	0.56 ± 0.53	0.018*
OCIPs	0.5 h	1.97 ± 2.72	0.929	1.15 ± 1.01	0.836	1.10 ± 1.03	0.709	1.28 ± 1.45	0.802
	2 h	0.90 ± 1.01	0.343	0.91 ± 1.19	0.399	1.11 ± 1.34	0.636	0.90 ± 0.98	0.335
	6 h	0.24 ± 0.83	0.088	0.55 ± 0.73	0.083	0.70 ± 1.41	0.277	0.97 ± 1.59	0.476
	24 h	0.34 ± 1.40	0.157	0.84 ± 0.91	0.246	0.56 ± 0.60	0.039*	0.88 ± 0.79	0.192
CM	0.5 h	1.20 ± 1.38	0.740	1.14 ± 1.37	0.666	1.33 ± 1.67	0.800	1.15 ± 1.66	0.637
	2 h	0.40 ± 0.60	0.029*	0.66 ± 0.62	0.018*	0.49 ± 0.53	0.012*	0.62 ± 0.50	0.001*
	6 h	0.70 ± 0.94	0.149	0.95 ± 0.92	0.374	0.71 ± 0.71	0.054	0.91 ± 1.12	0.362
	24 h	0.72 ± 0.85	0.117	1.04 ± 0.94	0.602	0.50 ± 1.22	0.144	0.71 ± 1.29	0.238

Significant results (p<0.05) are marked w ith asterisk*.

**Figure 3:**
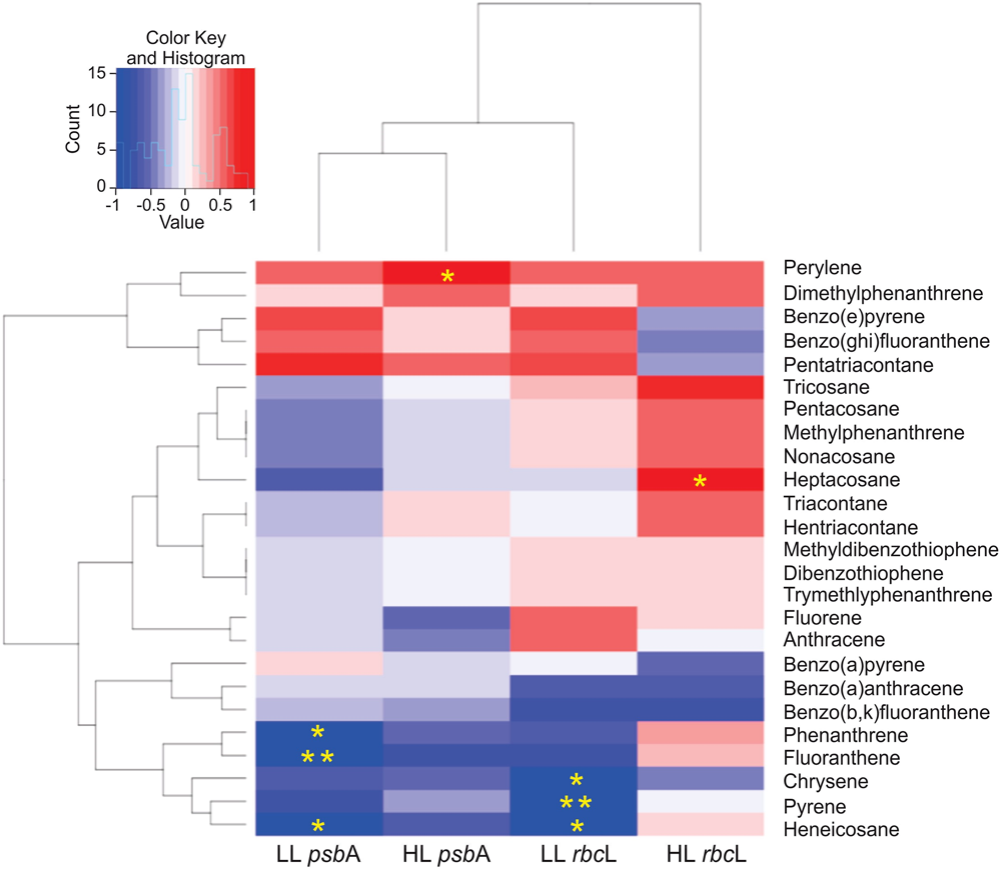
Correlations between changes in gene expression after 2 h exposure and the increase on concentration of the different components of the CM mixtures. Both magnitudes are expressed as fold changes (treated versus control). A, Spearman correlation heatmap. Significant correlations are indicated by asterisks (*p < 0.05; **p < 0.01). B) Double log plots between changes in *rbc*L LL and *psb*A LL mRNA levels (top and bottom X-axis, respectively) and in the concentration of different components (Y-axis), expressed both as fold changes (Treated versus Controls). Regression lines and the corresponding R^2^ values are indicated.

**Figure 4:**
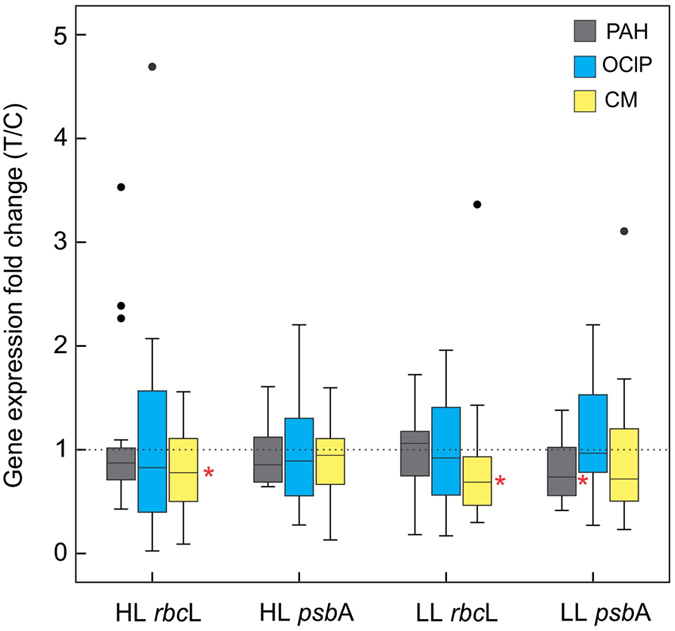
Distribution of gene expression changes (Treatment/Control) between the different Treated/Control samples for all treatments and incubation times. Significant differences (p-values) were calculated using paired t-tests (*p < 0.05, indicated on the right side).

The relative influence of the different components of the CM of OPs (the analyzed target OPs) on the expression of photosynthetic *Prochlorococcus* genes after two hours of incubation were studied by correlation analyses (Fig. 3). The analysis shows significant negative correlations between both LL genes and some PAHs with 3 and 4 aromatic rings: fluorene, phenanthrene, fluoranthene, pyrene, and chrysene, and the C21 alkane heneicosane (Fig. 3). These PAHs are predominant in the PAH pattern found in marine waters^2^ and they are surrogates of the overall SALCs pool in seawater. SALC concentrations in seawater are two or three orders of magnitude larger than that of individual PAHs^2^. The observed variation of LL *rbcL* and *psbA* when *Prochlorococcus* was exposed to CM are consistent with the negative effect of PAHs (alone but at higher concentrations) on the gene expression of LL genes (Fig. 4, Table 1). Nonetheless, our results suggest that it is the mixture of OPs at ultra-trace levels that is responsible for the observed inhibition of both HL and LL genes (Fig. 4, Table 1). This mixture of OPs included PAHs, but it has a major contribution of the aromatic UCM, made up of thousands of other aromatic-like hydrocarbons^2^. On the other hand, the observed positive correlation between gene expression and heptacosane and perylene concentration (Fig. 3) is consistent with the known biogenic sources of these compounds (cell wall waxes and diagenetic)^9, 22, 25^ and with the use of some hydrocarbons as a carbon source^9, 26^.

The exposure of *Prochlorococcus* to the PAH mixture alone resulted in a significant decrease of LL *psbA* mRNA levels at all incubation times (paired t-test, p < 0.01), except for the 2 h incubation, reaching the maximum effect (45% decrease) after 24 h of exposure. The LL expression values did not recover completely their initial levels after 24 h of exposure (Figs 2 and S1), even though the light conditions were similar than the initial ones. Although the available results do not allow for a rigorous analysis of this complex effect, it is likely that the presence of pollutants may contribute to increase the stress of LL cells under unfavorable conditions. No significant effects were observed when natural populations of *Prochlorococcus* were exposed to OClPs (Fig. 4).

Whereas the PAH incubation experiments were carried out at concentrations much higher than those found in the open ocean, the perturbation on gene expression in the CM treatments occurred at concentrations between 1.3 and 10 times those in controls (environmental concentrations). The concentrations of organic pollutants measured in the dissolved phase of the incubations with a CM reflect the general pattern of POPs, PAHs, n-alkanes and other hydrocarbons found in seawater, such as the UCM. The results obtained here confirm that a small perturbation of the concentrations of the thousands of OP entering seawater can induce a dysregulation of gene expression of higher magnitude than that due to few chemicals at higher concentrations. Therefore, there is probably a dose additivity in terms of effects for many of the individual chemicals in the CM, especially the aromatic compounds. For the same CM of organic pollutants (generated from the non-polar extract of seawater from the same region in the NE Atlantic), Echeveste and coworkers^3^ estimated that a 10% decrease of *Prochlorococcus* and *Synechococcus* abundance would be observed at C/C_Control_ values of 21 and 27, respectively, significantly higher than the factor 1.3–2 measured here. In any case, a dysregulation of the gene expression is likely to occur at significantly lower concentrations than those needed to cause a reduction of the abundance of cells^3^. Compounds such as n-alkanes and PAHs are among the most abundant resolved compounds in the called “aliphatic” and “aromatic” fractions of seawater (accounting for the non-polar fraction of the seawater extract used here). PAH and n-alkanes can be considered tracers (or surrogates) of the whole mixture of hydrocarbons and OPs, as these OPs have similar physical-chemical properties than the other compounds present in the same non-polar fraction of the seawater extract, and chemicals with similar properties behave similarly^19^. These results are not only consistent with those described for lethality effects on oceanic *Prochlorococcus* ^3, 27^, but also with other studies performed with different toxicity endpoints, in which the toxicity of known compounds is a small fraction of the total toxicity detected in the sample^20, 21^, and with other studies reporting the toxicity of the aromatic UCM^28, 29^.

Our findings emphasize the importance of the study of the effects of complex mixtures of organic pollutants instead assessing the effects of single compounds. This work also highlights the importance of performing studies using natural communities with an intrinsic variability of the different strains, and the impact of the incorporation of advanced, high sensitivity-high selectively analytical tools to the analysis of the anthropogenic impact on the Biosphere. The extrapolation of the results of these experiments to oceanic waters is difficult due to the multiple processes involved. It is clear from studies in different organisms that dysregulation of key genes is a powerful indicator of the presence of stressors in natural populations^30^, even though changes in transcript levels are not sufficient to predict protein levels or to constitute an adverse effect by themselves. Nevertheless, and since *rbcL* encodes the large subunit of RuBisCO, responsible of carbon fixation, and *psbA* encodes the core protein D1 of photosystem II, the primary target of photoinactivation, changes in their transcript levels could be used as indicators of alterations of the photosynthetic function.

While the photosynthetic capacity of *Prochlorococcus* natural populations obviously depends on many key variables such as nutrient availability, radiation and various environmental stressors, the action of pollutants remains as one of the less studied vectors of global change^1^, and one of the less studied stressors in phytoplankton ecology^31^. Our findings suggest that organic pollutants might co-modulate the photosynthetic function of *Prochlorococcus*, an issue requiring further research by means of experimental and field-scale work. The results reported here provide the first evidence that small variations (less than 2-fold) in seawater concentrations of complex mixtures of organic pollutants can induce an effect on the expression of *Prochlorococcus* photosynthetic genes, a variation considerably smaller than the one observed for the concentrations of OPs at the oceanic scale^2, 5–8^. Therefore, the combined effect of the myriad of organic pollutants present in the oceans may affect the carbon fluxes mediated by phytoplankton. This perturbation may contribute to the decline in phytoplankton abundance and productivity reported during the Anthropocene for some oceanic regions^32, 33^.

## Methods

### Experiments with natural *Prochlorococcus* communities

During the Malaspina circumnavigation cruise, from December 14^th^, 2010 to July 14^th^, 2011 on board the R/V Hesperides, we performed a total of 13 experiments in different locations of the Indian, Pacific and Atlantic Oceans (Fig. 1 and Table S6). Experiments were carried out by challenging natural populations of *Prochlorococcus* from the deep chlorophyll maximum (DCM) with three different mixtures of OPs (see below).

All the experiments started between 10 and 12:30 h local time, coinciding with the peak of carbon fixation by *Prochlorococcus*, which occurs between dawn and midday^34, 35^. The incubations were performed in baked 1-L glass bottles. The pollutant mixtures were added to the treatment bottles and the solvent (acetone) to the control bottles 1 hour before adding the seawater to allow for evaporation and avoid potential toxic effects of the solvents used to prepare the OPs mixtures. One liter of seawater from DCM was added, and the bottles immediately placed in an incubator located on the deck of the vessel that maintained the DCM temperature. The light radiation at the sampled depth was simulated using a net covering the bottles. Each experiment consisted of 4 different exposure times: 0.5 h, 2 h, 6 h and 24 h, using one pair of treatment and control for each time point. We collected samples to study *Prochlorococcus* gene expression at every exposure time following the protocol described below. Additional samples were taken at 0.5 h and 24 h to analyze the concentration of dissolved-phase OPs, and the picoplankton cell abundance by flow cytometry.

### Preparation of pollutant- spiked solutions

We used two simple mixtures of pollutants obtained from pure standards, and a complex mixture of pollutants directly obtained from seawater. The first simple mixture contained the 16 polycyclic aromatic hydrocarbons (PAHs) regulated by the US Environmental Protection Agency (EPA) (acenaphthene, acenaphthylene, anthracene, benzo(a)anthracene, benzo(a)pyrene, benzo(b)fluoranthene, benzo(g,h,i)perylene, benzo(k)fluoranthene, chrysene, dibenzo(a,h)anthracene, fluoranthene, fluorene, indeno(1,2,3-cd)pyrene, naphthalene, phenanthrene and pyrene) spiked at a nominal concentration of 700 ng/L, the approximated concentration estimated to reduce growth of natural populations of *Prochlorococcus* by 10% (LC10)^3^. No equivalent toxicity data exist for the second simple mixture containing organochlorine pesticides (OClPs), so it was spiked at a nominal concentration of 500 ng/L, which is known not to effect *Prochlorococcus* growth rates^18^. The OClPs mixture contained hexachlorobenzene (HCB) and the α, β, γ and δ isomers of hexaclorocyclohexane (HCH). Both PAHs and OClPs are ubiquitously found in oceanic waters and plankton^2, 4, 7, 36^.

The third pollutant spike solution was the non-polar fraction of a seawater extract containing every hydrophobic organic pollutant found in the surface ocean (4 m depth) at a nominal concentration of 10-fold their surface concentrations as measured in the NE Atlantic. It is referred in this work as the complex mixture (CM). Briefly, the CM was obtained by sampling oceanic seawater as usually performed for POPs analysis^2, 7, 36^. Briefly, 200 L of seawater were eluted and concentrated on a XAD-2 adsorbent after being filtered on a pre-combusted glass fiber filter (GFF, Whatmann). The sampling of seawater was performed in the NE Atlantic ocean^3^. XAD-2 columns were eluted sequentially with methanol and dichloromethane. The methanol fraction (containing seawater) was concentrated to 50 mL and liquid-liquid extracted with hexane in order to eliminate the residual water and the water soluble (polar) organic compounds. The hexane and dichloromethane fractions were merged and concentrated to 0.5 mL. This concentrated extract was then further fractionated on an alumina column to separate the polar and non-polar compounds. The latter fraction, eluted with hexane and hexane:dichloromethane (3:1), contains most of the chemicals with properties typical of POPs, such as PCBs, PAHs and all other aliphatic and aromatic hydrocarbons including the UCMs, OClPs, polybrominated diphenyl ethers, polychlorinated dibenzo-p-dioxins and dibenzofurans, dechlorane plus, chlorinated naphthalenes, among other OPs that have been previously described in the marine environment^19^. This CM mixture is the most non-polar fraction of the dissolved OC (O/C ratio lower than 0.1, but even 0 for most compounds), as the polar compounds which dominate in mass the dissolved organic carbon pool have been eliminated through the triple fractionation steps described above: (i) extraction of seawater with the hydrophobic XAD-2 amberlite, (ii) extraction of the polar extract of XAD with hexane, (iii) fractionation on an Alumina column. An exploratory analysis of the chemicals comprising the aromatic fraction has been described elsewhere^2^, and those from the aliphatic fraction show obviously even lower polarity. The non-polar chemical mixture obtained following this methodology also contain unknown OPs which have not been described yet in the literature due to lack of appropriate analytical methods^37^, and which can contribute significantly to the overall CM toxicity. In any case, the unresolved complex mixture comprises most of the mass contributing to this CM^2^. Volatile organic compounds (operationally defined with higher vapor pressures than naphthalene and dodecane) were not present in the CM as the most volatile compounds were lost during the preparation of the mixture. The concentrations in the CM of PAHs, PCBs and OClPs were measured prior to their use in the experiments, using the methods described elsewhere^6, 27, 36^, and the amount of each contaminant added to the treatments is reported in Table S4. The added amount of CM added to the 1 L bottles used in the incubations are those corresponding to 10 L of seawater from the NE Atlantic, and thus the nominal enrichment for the experiments with CM was of C/C_Control_ = 10. The solvents used during the preparation of the extracts were always of the highest purity available with the targeted OPs bellow the limits of quantification. The blanks of the XAD-2 columns and fractionation schemes lead to levels of organic pollutants, such as PAHs and PCBs, bellow the limits of detection in most cases, or significantly lower than those in seawater samples as reported elsewhere^2, 10^. The levels of the UCM in the blanks were below detection limits.

### **A**nalysis of dissolved phase concentrations of organic pollutants

In addition of the analysis performed prior to the use of the CM in the experiments, the organic pollutants were also measured at the start and at the end of the experiments. After sample collection for gene expression analyses by filtering onto a 0.2-μm pore-size filter, we added to the filtered water a surrogate standard mix containing five deuterated PAHs (naphtalene-d10, acenaphthene-d10, phenanthrene-d10, chrysene-d12 and perylene-d12), one deuterated n-alkane (tetracosane-d50) and two PCBs (PCB 65 and PCB 200). The filtered water was then pre-concentrated in a solid-phase extraction 6 cc Oasis HLB cartridge (Waters, Montevideo, Uruguay) containing 500 mg of sorbent, using a vacuum manifold. The SPE cartridges had been previously conditioned with 5 mL of hexane followed by 5 mL of dichloromethane/hexane (2:1), 5 mL of dichloromethane/methanol (2:1) and 5 mL of HPLC-grade water. The Oasis cartridges were eluted with 5 mL of hexane, 10 mL of dichloromethane/hexane (2:1), and 5 mL of dichloromethane/methanol (2:1). Any aqueous residual in the extract was purified on a glass funnel filled with 50–60 g of anhydrous sodium sulfate. The extract was concentrated to 0.5–1 mL by vacuum rotary evaporation, transferred to a 1.5 mL amber vial, and evaporated to 50–100 μL under a gentle nitrogen stream. We analyzed a number of target organic pollutants in the water from the experiments performed with CM: PAHs, n-alkanes, organochlorine pesticides (HCHs and HCB) and PCBs. These OPs cover a wide range of physical chemical properties (solubility, vapor pressure, hydrophobicity, and persistence) and are used here as surrogates of the OPs present in seawater, as it is unfeasible to analyze all of them. In the experiments challenged with the simple mixtures of PAHs and OClPs, we analyzed only the family of pollutants that was fortified. Before the instrumental analysis for the quantification of PAHs, n-alkanes and PCBs, we added to the extract an internal standard mix containing 50 ng of anthracene-d10, pyrene-d10, P-therphenyl-d14 and benzo(b)fluoranthene-d12; 12 µg of nonadecane-d40; and 2 ng of the PCBs congeners 30 and 142. PAHs and n-alkanes were quantified by gas-chromatography coupled to mass spectrometry, and PCBs, HCB and HCHs by gas-chromatography coupled to an electron capture detector as described elsewhere^2, 6, 7, 36^.

### **C**ell abundance estimation

The cell concentrations of *Prochlorococcus*, *Synechococcus* and picoeukaryotes were measured *in vivo* on board of the ship in subsamples of 1 mL by flow cytometry using a FACSCalibur (Becton Dickinson Biosciences, San Jose, California, USA) as explained elsewhere^38^.

### *Prochlorococcus* gene expression analyses

Sample collection and analyzes were performed using the procedures described elsewhere^18^. 990 mL of seawater from the incubations were filtered onto 47-mm-diameter, 0.2-μm pore-size PTFE filters (Millipore, Billerica, MA) under low vacuum pressure. Each filter was split into two halves, one was placed into RNAlater (Sigma-Aldrich, Saint Louis, MO) at −80 °C to preserve RNA, and the other one into lysis buffer (50 mM Tris HCl, 40 mM EDTA, 0.75 M Sucrose) at −20 °C to preserve DNA^18^.

For RNA isolation, we extracted the half-filter samples in RNAlater using the mirVana™ kit (Ambion, Austin, TX), after removing the storage reagent by centrifugation. We concentrated total RNA by partial lyophilization to approximately 40 µL, measured its concentration by a NanoDrop ND_8000 spectrophotometer (NanoDrop Technologies, Delaware, DE). Quality was checked with an Agilent 2100 Bioanalyzer (Agilent Technologies, Santa Clara, CA). Total RNA was treated with DNase I (Ambion) to remove genomic DNA contamination and reverse transcribed to cDNA using First Strand cDNA Synthesis Kit (Roche, Mannheim, GE). The resulting cDNA preparation was stored at −80 °C until quantitative real-time PCR (qRT-PCR) analysis was performed.

The target genes selected were *rbcL*, which encodes the large subunit of RuBisCO, responsible of carbon fixation, and *psbA*, that encodes the core protein D1 of photosystem II, the primary target of photoinactivation. To normalize the expression of these genes we used *rnpB* as reference gene, which encodes the RNA component of the RNase P, and that has been described as a suitable reference gene for qRT-PCR analysis^18, 39–41^. Specific sets of primers were designed to target HL and LL *Prochlorococcus* clades’ genes separately, as described elsewhere^18^.

Aliquots of 3.75 ng of total cDNA were amplified by qRT-PCR in a LightCycler 480 (Roche Diagnostics, Indianapolis, IN) thermocycler using SYBR Green Mix (Takara Bio Inc., Siga, Japan) with the following parameters: activation at 95 °C for 10 s, forty-five amplification cycles (95 °C for 5 s, 60 °C for 35 s), followed by a melting curve program (65–95 °C with a heating rate of 0.11 °C /s) and a final extension at 60 °C for 30 s. All samples were run in duplicates, and the amplifications of target and reference genes of the same sample were performed in the same plate in order to minimize systematic errors.

We used the second derivate maximum of the amplification curves (Cp) to calculate the relative quantity of mRNA of each gene. Cp values for the target genes (tg) *rbcL* and *psbA* were normalized to reference gene *rnpB* to obtain ∆Cp values as explained elsewhere^18^. The ratios between treatments and controls mRNA/DNA levels were calculated from these ∆Cp values, as Copies_Treatment_/Copies_Control_ = 2^(∆CpTreatment − ∆CpControl)^ ^18^. The PCR efficiency for the tested genes were calculated as described elsewhere^42^.

### Statistical analysis

Given the experimental design, in which each experiment started from a freshly obtained DCM water sample split into pairs of independent containers, one treated and the other left as control, comparisons between treated and untreated cultures were performed using paired t-test assays for both qRT-PCR and OP concentrations. Time variations were analyzed using the non-parametric Kruskal-Wallis test, as it is more robust against outliers than the parametric ANOVA counterpart. Comparisons between T/C (treatments versus control) ratios for qRT-PCR or pollutant concentrations were also analyzed by non-parametric (Spearman’s) tests, to account for their intrinsic non-linearity and their different data structure. Concentrations of organic pollutants in the dissolved phase presenting values below the method detection limit (MDL) were replaced with MDL/2, and missing data values were substituted with median concentrations. Statistical tests and figures were performed using the SPSS 19 (SPSS Inc., Chicago, IL) package. Figures were edited using CorelDraw × 6 (Corel Corporation, Ottawa, Ontario, Canada). Additional statistical analyses (e.g., heatmap) were performed using the R package (http://CRAN.R-project.org/).

### Availability of Data

All datasets generated and/or analysed during the current study are given in the supplementary material or available from the corresponding author on reasonable request.

## Electronic supplementary material

Supplementary Information
