# Supplementary material for: Dysregulation of photosynthetic genes in oceanic Prochlorococcus populations exposed to organic pollutants
Source: Sci Rep. 2017 Aug 14;7:8029. doi: 10.1038/s41598-017-08425-9 (PMC5556013; doi:10.1038/s41598-017-08425-9)
Supplement: Supplementary file 1 — Supplementary Information [file 41598_2017_8425_MOESM1_ESM.pdf]

**Supporting Information**

# Dysregulation of photosynthetic genes in oceanic *Prochlorococcus* populations exposed to organic pollutants

**Maria-Carmen Fernández-Pinos[1], Maria Vila-Costa[1], Jesús M. Arrieta[2,3], Laura Morales[1], Belén González-Gaya[1], Benjamí Piña[1], Jordi Dachs[1*]**

[1] Department of Environmental Chemistry, IDAEA-CSIC, Barcelona, Catalunya, Spain.

[2] Spanish Institute of Oceanography (IEO), Oceanographic Center of The Canary Islands, Santa Cruz de Tenerife, 38180, Spain

[3] Department of Global Change Research. IMEDEA-UIB-CSIC, Esporles, Mallorca, Spain.

*Corresponding author email: jordi.dachs@idaea.csic.es

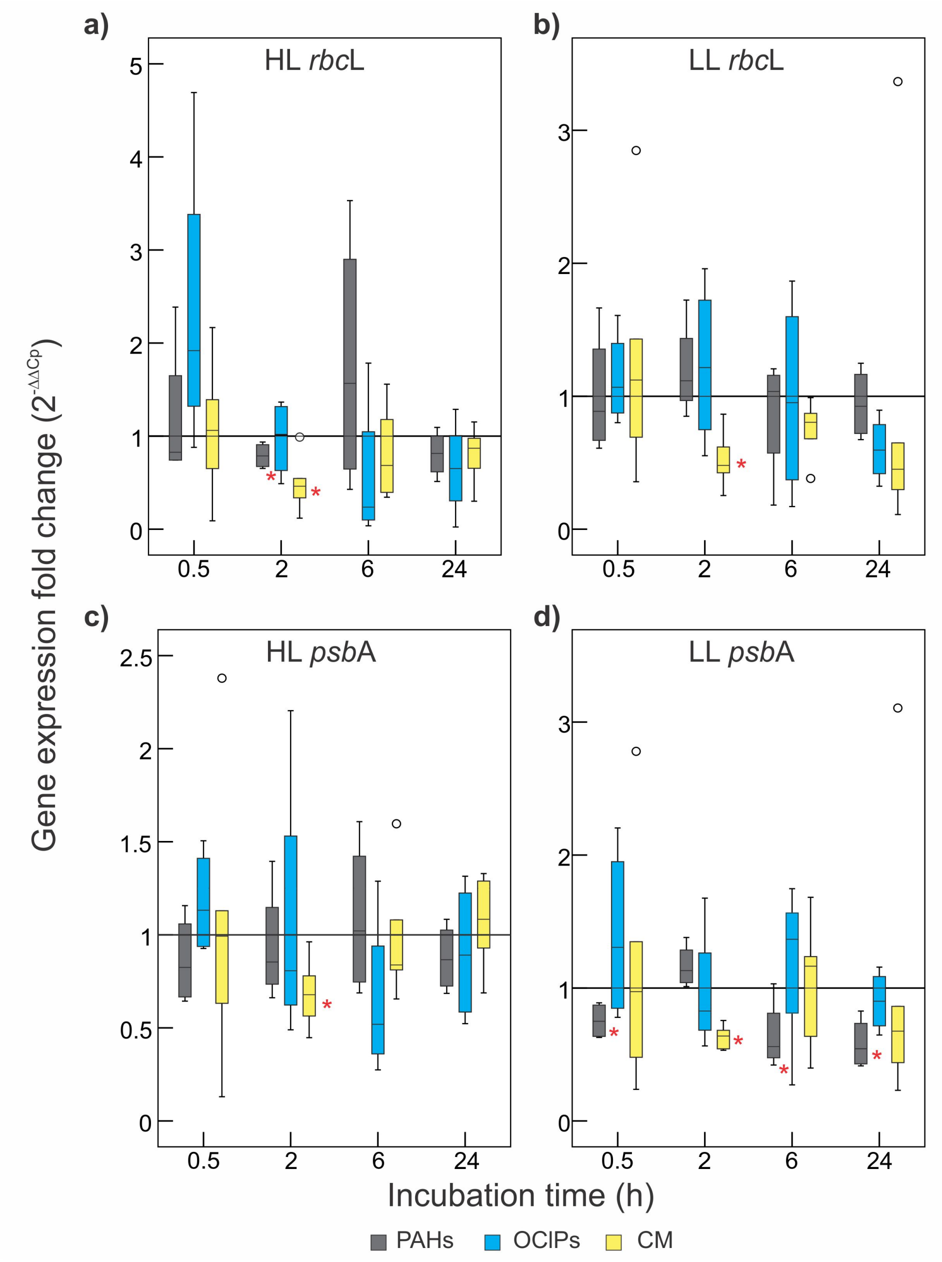


Figure S1. Gene expression of *Prochlorococcus rbc*L (a,b) and *psb*A (c,d) genes of treatments and controls were analysed by paired t-tests for the four incubation times tested. LL *Prochlorococcus* (b, d) showed more significant effects of pollutants (p-value < 0.05 are marked with "*") than HL *Prochlorococcus* (a,c). The most toxic pollutant mixture was the complex mixture (CM, yellow), that produced a significant decrease of LL *rbc*L (b) and *psb*A expression from both clades (c,d) after 2 h of exposure. In contrast, PAH

mixture (grey) only affected significantly the expression of LL psbA after 0.5 h and 24 h (d), and the OClP mixture that did not affect significantly gene expression of any target gene.

Table S1. Relative concentrations (C/Ccontrol) of PAHs measured in the PAH experiments after 0.5 h and 24 h of incubation

| | PAH_Ind | | PAH_Pac1 | | PAH_Pac2 | | PAH_Atl | | **Average** | |
|---|---|---|---|---|---|---|---|---|---|---|
| | 0.5 h | 24 h | 0.5 h | 24 h | 0.5 h | 24 h | 0.5 h | 24 h | 0.5 h | 24 h |
| Fluorene | 12.4 | 9.5 | 16.7 | 7.6 | 45.1 | 13.9 | 22.6 | 39.4 | 24.2 | 17.6 |
| Phenanthrene | 20.0 | 32.8 | 9.6 | 16.5 | 37.2 | 24.5 | 18.1 | 56.3 | 21.2 | 32.5 |
| Antracene | 47.7 | 9.7 | 123 | 9.9 | 394 | 2.9 | 211 | 29.7 | 194 | 13.1 |
| Fluoranthene | 17.4 | 57.8 | 103 | 299 | 257 | 65.7 | 159 | 188 | 134 | 153 |
| Pyrene | 15.6 | 43.6 | 161 | 173 | 351 | 82.5 | 93 | 71 | 155 | 92 |
| Benzo(a)anthracene | 22.4 | 4.5 | 317 | 178 | 488 | 33.3 | 800 | 249 | 407 | 116 |
| Chrysene | 59.5 | 6.7 | 280 | 338 | 371 | 74.8 | 681 | 458 | 348 | 220 |
| Benzo(byk)fluoranthene | 19.5 | 4.6 | 48.5 | 18.8 | 27.1 | 3.7 | 24.4 | 11.4 | 29.9 | 9.6 |
| Benzo(a)pyrene | 74.2 | 13.7 | 51.3 | 12.9 | 64.0 | 2.5 | 41.3 | 8.3 | 57.7 | 9.3 |
| **Average** | 32.1 | 20.3 | 123 | 117 | 226 | 33.8 | 228 | 124 | 152 | 74 |

Table S2. Relative concentrations (C/Ccontrol) of HCB and HCH isomers measured in the OCIP experiments after 0.5 h and 24 h of incubation.

| | OCIP_Pac1 | | OCIP_Pac2 | | OCIP_Pac3 | | OCIP_Atl | | **Average** | |
|---|---|---|---|---|---|---|---|---|---|---|
| | 0.5 h | 24 h | 0.5 h | 24 h | 0.5 h | 24 h | 0.5 h | 24 h | 0.5 h | 24 h |
| HCB | 1.0 | 1.1 | 19 | N/A | 11 | 23 | 21 | 19 | 13 | 14 |
| αHCH | 5.0 | 6.4 | 62 | N/A | 27 | 32 | 71 | 124 | 41 | 54 |
| γHCH | 734 | 1067 | 1341 | N/A | 1109 | 880 | 691 | 1033 | 969 | 993 |
| βHCH | 233 | 164 | N/D | N/A | N/D | 459 | N/D | N/D | 233 | 311 |
| δHCH | 827 | 483 | N/D | N/A | N/D | N/D | N/D | N/D | 827 | 483 |
| **Average** | 360 | 344 | 474 | N/A | 382 | 348 | 261 | 392 | 369 | 362 |

Table S3. Relative concentrations (C/Ccontrol) of PAHs and Alkanes measured in the complex mixtures experiments as surrogate after 0.5 h and 24 h of incubation. N/A = Not available, N/D = compound non detected in the control.

| | | CM_Ind | | CM_Pac1 | | CM_Pac2 | | CM_Atl1 | | CM_Atl2 | | **Average** | |
|---|---|---|---|---|---|---|---|---|---|---|---|---|---|
| | | 0.5 h | 24 h | 0.5 h | 24 h | 0.5 h | 24 h | 0.5 h | 24 h | 0.5 h | 24 h | 0.5 h | 24 h |
| PAHs | Fluorene | 1.3 | 1.3 | 1.1 | 1.3 | 0.9 | 0.8 | 0.7 | 1.8 | 0.8 | 1.0 | 1.0 | 1.2 |
| | Dibenzothiophene | 1.2 | 1.2 | 0.9 | 1.3 | 0.9 | 1.7 | 1.5 | 1.4 | 0.7 | 0.7 | 1.0 | 1.3 |
| | Methyldibenzothiophene | 1.2 | 1.2 | 0.9 | 1.4 | 0.5 | 1.7 | 1.4 | 1.4 | 0.4 | 0.8 | 0.9 | 1.3 |
| | Phenanthrene | 1.5 | 1.5 | 0.8 | 1.2 | 0.9 | 1.0 | 1.3 | 2.2 | 1.0 | 1.0 | 1.1 | 1.4 |
| | Methylphenanthrene | 1.3 | 1.3 | 1.0 | 1.4 | 1.1 | 1.7 | 1.3 | 1.5 | 0.5 | 0.8 | 1.0 | 1.3 |
| | Dimethylphenanthrene | 1.1 | 1.1 | 0.9 | 1.3 | 1.1 | 1.4 | 1.7 | 1.6 | 0.2 | 0.6 | 1.0 | 1.2 |
| | Trymethylphenanthrene | 1.1 | 1.1 | 1.1 | 0.7 | 0.6 | 1.9 | 1.3 | 1.3 | 0.3 | 1.0 | 0.8 | 1.2 |
| | Anthracene | 1.4 | 1.4 | 1.2 | 1.3 | 1.0 | 1.1 | 1.0 | 3.3 | 0.8 | 0.7 | 1.1 | 1.6 |
| | Fluoranthene | 2.0 | 2.0 | 0.6 | 1.5 | 1.1 | 1.1 | 1.3 | 1.5 | 1.7 | 0.9 | 1.3 | 1.4 |
| | Pyrene | 1.4 | 1.4 | 0.6 | 1.5 | 1.1 | 1.0 | 1.5 | 0.3 | 7.9 | 0.9 | 2.5 | 1.0 |
| | Benzo(ghi)fluoranthene | 1.1 | 1.1 | 1.4 | 0.9 | 0.3 | 2.3 | 1.2 | 1.1 | 0.3 | 1.0 | 0.8 | 1.3 |
| | Benzo(a)anthracene | 1.1 | 1.1 | 1.2 | 0.9 | 1.0 | 0.6 | 2.2 | 1.9 | 2.0 | 0.9 | 1.5 | 1.1 |
| | Chrysene | 1.1 | 1.1 | 1.1 | 0.9 | 0.9 | 0.3 | 3.4 | 2.4 | 6.1 | 0.9 | 2.5 | 1.1 |
| | Benzo(byk)fluoranthene | 1.1 | 1.1 | 1.1 | 0.7 | 0.7 | 1.7 | 1.3 | 4.1 | 2.2 | 1.0 | 1.3 | 1.7 |
| | Benzo(e)pyrene | 1.0 | 1.0 | 1.3 | 1.0 | 1.2 | 1.7 | 1.0 | 1.1 | 0.0 | 1.0 | 0.9 | 1.2 |
| | Benzo(a)pyrene | 1.3 | 1.3 | 1.4 | 0.9 | 0.5 | 1.1 | 1.6 | 0.9 | 0.7 | 1.0 | 1.1 | 1.1 |
| | Perylene | 0.9 | 0.9 | 1.0 | 0.7 | 1.0 | 2.7 | 1.0 | 1.1 | 0.3 | 0.8 | 0.8 | 1.2 |
| | **Average** | **1.2** | **1.2** | **1.0** | **1.1** | **0.9** | **1.4** | **1.4** | **1.7** | **1.5** | **0.9** | **1.2** | **1.3** |
| Alkanes | Heneicosane | 4.5 | 4.5 | 1.2 | 2.2 | 3.4 | 1.0 | 3.5 | 2.0 | 15.7 | 1.7 | 5.7 | 2.3 |
| | Tricosane | 5.9 | 5.9 | 1.1 | 1.9 | 2.4 | 1.5 | 2.3 | 0.8 | 0.7 | 1.1 | 2.5 | 2.3 |
| | Pentacosane | 5.1 | 5.1 | 1.1 | 1.7 | 2.3 | 1.9 | 2.4 | 0.7 | 0.4 | 0.5 | 2.3 | 2.0 |
| | Heptacosane | 7.9 | 7.9 | 1.1 | 1.6 | 2.2 | 1.4 | 2.0 | 0.9 | 1.1 | 1.3 | 2.9 | 2.6 |
| | Nonacosane | 3.7 | 3.7 | 1.1 | 1.5 | 1.5 | 1.3 | 2.0 | 0.9 | 0.1 | 1.2 | 1.7 | 1.7 |
| | Hentriacontane | 1.8 | 1.8 | 1.0 | 1.0 | 1.1 | 1.1 | 1.8 | 1.2 | 0.2 | 1.2 | 1.2 | 1.3 |
| | Tritriacontane | 1.2 | 1.2 | 0.9 | 1.1 | 0.9 | 0.8 | 1.5 | 1.0 | 0.8 | 2.0 | 1.1 | 1.2 |
| | Pentatriacontane | 1.0 | 1.0 | 2.0 | 2.0 | 1.0 | 1.0 | 1.0 | 1.0 | 0.9 | 1.0 | 1.2 | 1.2 |
| | **Average** | **3.9** | **3.9** | **1.2** | **1.6** | **1.8** | **1.3** | **2.1** | **1.1** | **2.5** | **1.3** | **2.3** | **1.8** |

Table S4. Amounts of the measured organic pollutants that were added to the seawater in the experiments challenged with complex mixtures of pollutants.

| | Organic pollutant | Amount of pollutant added to 1L of experimental sample (pg) | | | | | |
|---|---|---|---|---|---|---|---|
| | | CM_Ind | CM_Pac1 | CM_Pac2 | CM_Atl1 | CM_Atl2 | **Average** |
| Organochlorinated Pesticides | HCB | 0.39 | 7.60 | 7.60 | 9.03 | 9.03 | 8.31 |
| | α-HCH | 0.00 | 1.11 | 1.11 | 1.54 | 1.54 | 1.32 |
| | γ-HCH | 0.07 | 0.43 | 0.43 | 0.00 | 0.00 | 0.22 |
| | δ-HCH | 0.04 | 1.11 | 1.11 | 2.29 | 2.29 | 1.70 |
| DDT related compounds | 4,4-DDD | 0.11 | 1.78 | 1.78 | 3.96 | 3.96 | 2.87 |
| | 4,4'-DDD | 0.00 | 1.63 | 1.63 | 4.71 | 4.71 | 3.17 |
| | 2,4'-DDT | 0.00 | 1.06 | 1.06 | 2.86 | 2.86 | 1.96 |
| | 4,4'-DDT | 0.08 | 4.57 | 4.57 | 7.44 | 7.44 | 6.01 |
| Polychlorinated Biphenyls (PCBs) | PCB 18 | 0.28 | 3.03 | 3.03 | 0.00 | 0.00 | 1.51 |
| | PCB 17 | 0.04 | 0.38 | 0.38 | 0.00 | 0.00 | 0.19 |
| | PCB 31 | 0.31 | 1.78 | 1.78 | 2.56 | 2.56 | 2.17 |
| | PCB 28 | 0.28 | 2.69 | 2.69 | 3.74 | 3.74 | 3.22 |
| | PCB 33 | 0.00 | 2.69 | 2.69 | 5.77 | 5.77 | 4.23 |
| | PCB 52 | 0.26 | 2.79 | 2.79 | 14.67 | 14.67 | 8.73 |
| | PCB 49 | 0.20 | 2.12 | 2.12 | 0.00 | 0.00 | 1.06 |
| | PCB 44 | 0.00 | 31.01 | 31.01 | 0.00 | 0.00 | 15.50 |
| | PCB 74 | 0.00 | 1.73 | 1.73 | 0.00 | 0.00 | 0.87 |
| | PCB 70 | 0.20 | 3.27 | 3.27 | 3.66 | 3.66 | 3.46 |
| | PCB 95 | 0.37 | 6.68 | 6.68 | 9.25 | 9.25 | 7.97 |
| | PCB 99+PCB 1 | 0.81 | 13.46 | 13.46 | 10.22 | 10.22 | 11.84 |
| | PCB 87 | 0.16 | 1.73 | 1.73 | 5.68 | 5.68 | 3.71 |
| | PCB 110 | 0.17 | 2.40 | 2.40 | 3.39 | 3.39 | 2.90 |
| | PCB 82 | 0.16 | 3.41 | 3.41 | 5.77 | 5.77 | 4.59 |
| | PCB 151 | 0.00 | 2.79 | 2.79 | 0.00 | 0.00 | 1.39 |
| | PCB 149 | 0.23 | 7.26 | 7.26 | 7.93 | 7.93 | 7.59 |
| | PCB 118 | 0.30 | 8.75 | 8.75 | 14.41 | 14.41 | 11.58 |
| | PCB 153 | 0.31 | 9.33 | 9.33 | 13.52 | 13.52 | 11.43 |
| | PCB 132 | 0.14 | 2.07 | 2.07 | 3.48 | 3.48 | 2.77 |
| | PCB 105 | 0.08 | 1.20 | 1.20 | 3.61 | 3.61 | 2.41 |
| | PCB 138 | 0.36 | 6.92 | 6.92 | 3.08 | 3.08 | 5.00 |
| | PCB 158 | 0.04 | 1.06 | 1.06 | 5.07 | 5.07 | 3.06 |
| | PCB 187 | 0.19 | 4.04 | 4.04 | 0.00 | 0.00 | 2.02 |
| | PCB 183 | 0.00 | 1.54 | 1.54 | 0.00 | 0.00 | 0.77 |
| | PCB 177 | 0.00 | 0.72 | 0.72 | 0.00 | 0.00 | 0.36 |
| | PCB 171+PCB 15 | 0.00 | 1.39 | 1.39 | 1.32 | 1.32 | 1.36 |
| | PCB 180 | 0.11 | 3.03 | 3.03 | 14.89 | 14.89 | 8.96 |
| | PCB 191 | 0.00 | 6.01 | 6.01 | 0.00 | 0.00 | 3.00 |
| | PCB 170 | 0.00 | 0.91 | 0.91 | 1.01 | 1.01 | 0.96 |
| | PCB 201+PCB 19 | 0.00 | 2.07 | 2.07 | 0.00 | 0.00 | 1.03 |
| | PCB 195 | 0.04 | 0.48 | 0.48 | 0.44 | 0.44 | 0.46 |
| | PCB 194 | 0.00 | 0.48 | 0.48 | 0.79 | 0.79 | 0.64 |
| Polycyclic Aromatic Hidrocarbons (PAHs) | Fluorene | 21185 | 7062 | 7062 | 17654 | 17654 | 7062 |
| | Dibenzothiophene | 3068 | 1023 | 1023 | 2556 | 2556 | 1023 |
| | Metyldibenzothiopene | 8722 | 2907 | 2907 | 7268 | 7268 | 2907 |
| | Phenanthrene | 28705 | 9568 | 9568 | 23920 | 23920 | 9568 |
| | Anthracene | 3621 | 1207 | 1207 | 3018 | 3018 | 1207 |
| | Metylphenanthrene | 18907 | 6302 | 6302 | 15756 | 15756 | 6302 |
| | Dimetylphenanthrene | 9390 | 3130 | 3130 | 7825 | 7825 | 3130 |
| | Fluoranthene | 31562 | 10521 | 10521 | 26301 | 26301 | 10521 |
| | Pyrene | 22438 | 7479 | 7479 | 18699 | 18699 | 7479 |
| | Benzo[a]anthracene | 758 | 253 | 253 | 632 | 632 | 253 |
| | Crysene | 1409 | 470 | 470 | 1174 | 1174 | 470 |
| | Benzo[b]fluoranthene | 242 | 81 | 81 | 202 | 202 | 81 |
| | Benzo[k]fluoranthene | 83 | 28 | 28 | 69 | 69 | 28 |
| | Benzo[e]pyrene | 115 | 38 | 38 | 96 | 96 | 38 |
| | Benzo[a]pyrene | 99 | 33 | 33 | 83 | 83 | 33 |
| | Perylene | 44 | 15 | 15 | 37 | 37 | 15 |
| | Indeno[1,2,3-cd]pyrene | 1058 | 353 | 353 | 882 | 882 | 353 |
| | Dibenzo[a,h]anthracen | 1028 | 343 | 343 | 857 | 857 | 343 |
| | Benzo[ghi]perylene | 379 | 126 | 126 | 316 | 316 | 126 |

Table S5. Flow cytometer results for cyanobacteria and picoeukaryotes for the different experiment samples after 0.5 and 24 h on deck.

| Experiment | | Time (h) | Cell Counts (cell/mL) | | | | | | | | |
|---|---|---|---|---|---|---|---|---|---|---|---|
| | | | *Prochlorococus* | | | *Synechococcus* | | | Picoeukaryotes | | |
| | | | Control sample | Treated sample | p-value (T/C)[1] | Control sample | Treated sample | p-value (T/C)[1] | Control sample | Treated sample | p-value (T/C)[1] |
| PAHs | 29-May-11 | 0.5 | 2.14E+05 | 3.17E+05 | 0.615 | 1.46E+03 | 1.69E+03 | 0.780 | 1.92E+04 | 2.25E+04 | 0.638 |
| | 29-May-11 | 24 | 2.51E+04 | 1.29E+04 | | 1.78E+03 | 1.84E+03 | | 5.60E+03 | 1.25E+03 | |
| | 28-Jun-11 | 0.5 | 1.70E+04 | 2.36E+03 | | 6.59E+02 | 5.22E+02 | | 1.97E+05 | 1.96E+05 | |
| | 28-Jun-11 | 24 | 1.48E+05 | 1.47E+05 | | 1.75E+03 | 1.46E+03 | | 3.57E+03 | 3.07E+03 | |
| OCIP | 31-May-11 | 0.5 | 3.50E+05 | 3.66E+05 | 0.458 | 2.03E+04 | 2.14E+04 | 0.207 | 1.83E+04 | 1.85E+04 | 0.484 |
| | 31-May-11 | 24 | 2.54E+05 | 2.64E+05 | | 1.91E+04 | 2.31E+04 | | 1.17E+04 | 1.14E+04 | |
| | 25-Jun-11 | 0.5 | ND | ND | | 2.56E+03 | ND | | 1.74E+03 | ND | |
| | 25-Jun-11 | 24 | 1.37E+05 | 1.30E+05 | | 4.81E+03 | 5.46E+03 | | 3.49E+03 | 3.34E+03 | |
| CM | 5-Jun-11 | 0.5 | 8.85E+05 | 7.20E+04 | 0.322 | 8.59E+05 | 8.41E+05 | 0.395 | 1.04E+03 | 1.04E+05 | 0.348 |
| | 5-Jun-11 | 24 | 9.99E+04 | 6.95E+04 | | 1.12E+06 | 1.29E+06 | | 1.31E+05 | 1.39E+05 | |
| | 7-Jun-11 | 0.5 | 1.40E+05 | 1.50E+05 | | 7.43E+05 | 7.55E+05 | | 1.90E+05 | 1.89E+05 | |
| | 7-Jun-11 | 24 | 1.09E+05 | 1.20E+05 | | 4.69E+05 | 4.64E+05 | | 1.09E+05 | 1.03E+05 | |
| | 2-Jul-11 | 0.5 | 2.00E+05 | 2.07E+05 | | 3.91E+02 | 3.52E+02 | | 7.81E+03 | 6.80E+03 | |
| | 2-Jul-11 | 24 | 1.09E+05 | 1.14E+05 | | 6.40E+02 | 2.64E+02 | | 7.18E+03 | 4.29E+03 | |
| | 10-Jul-11 | 0.5 | 2.52E+05 | 1.17E+05 | | 6.35E+02 | 7.08E+02 | | 1.15E+05 | 6.12E+03 | |

1) Paired t-test, control vs. treated
ND, no data available

Table S6. Position and depth of the stations where the natural phytoplankton communities where sampled, date and local time of sampling, and type of pollutant mixture used to perform each experiment. PAH = polyaromatic hidrocarbon mixture; OClP = Organochlorinated pesticide mixture; and CM = complex mixture

| Name | Date | Pollutants | Ocean | Latitude | Longitude | DCM depth (m) |
|---|---|---|---|---|---|---|
| PAH_Ind | 1-Mar-11 | PAH | Indian | -29.892 | 76.066 | 140 |
| CM_Ind | 9-Mar-11 | CM | Indian | -30.327 | 103.32 | 100 |
| OClP_Pac1 | 28-Mar-11 | OClP | South Pacific | -38.699 | 150.436 | 60 |
| PAH_Pac1 | 16-May-11 | PAH | North Pacific | 21.063 | -150.442 | 105 |
| OClP_Pac2 | 19-May-11 | OClP | North Pacific | 19.917 | -141.635 | 125 |
| PAH_Pac2 | 29-May-11 | PAH | North Pacific | 13.187 | -113.267 | 70 |
| OClP_Pac3 | 31-May-11 | OClP | North Pacific | 11.978 | -108.032 | 59 |
| CM_Pac1 | 5-Jun-11 | CM | North Pacific | 8.809 | -93.143 | 24 |
| CM_Pac2 | 7-Jun-11 | CM | North Pacific | 7.207 | -87.9 | 20 |
| OClP_Atl | 25-Jun-11 | OClP | North Atlantic | 17.427 | -59.833 | 90 |
| PAH_Atl | 28-Jun-11 | PAH | North Atlantic | 20.014 | -52.691 | 130 |
| CM_Atl1 | 2-Jul-11 | CM | North Atlantic | 23.766 | -41.918 | 130 |
| CM_Atl2 | 10-Jul-11 | CM | North Atlantic | 32.084 | -17.286 | 110 |
